# Development and therapeutic evaluation of 5D3(CC-MLN8237)_3.2_ antibody-theranostic conjugates for PSMA-positive prostate cancer therapy

**DOI:** 10.3389/fphar.2024.1385598

**Published:** 2024-05-01

**Authors:** Ioanna Liatsou, Betelhem Assefa, Wathsala Liyanage, Sharmane Surasinghe, Zora Nováková, Cyril Bařinka, Kathleen Gabrielson, Venu Raman, Dmitri Artemov, Sudath Hapuarachchige

**Affiliations:** ^1^ Department of Radiology and Radiological Science, The Johns Hopkins University School of Medicine, Baltimore, MD, United States; ^2^ Department of Ophthalmology, The Johns Hopkins University School of Medicine, Baltimore, MD, United States; ^3^ Department of Neuroscience, Johns Hopkins University, Baltimore, MD, United States; ^4^ Laboratory of Structural Biology, Institute of Biotechnology of the Czech Academy of Sciences, Vestec, Czechia; ^5^ Department of Molecular and Comparative Pathobiology, The Johns Hopkins University School of Medicine, Baltimore, MD, United States; ^6^ Department of Oncology, The Sidney Kimmel Comprehensive Cancer Center, The Johns Hopkins University School of Medicine, Baltimore, MD, United States; ^7^ Department of Pathology, University Medical Center Utrecht, Utrecht, Netherlands; ^8^ Department of Pharmacology and Molecular Sciences, The Johns Hopkins University School of Medicine, Baltimore, MD, United States

**Keywords:** prostate cancer, PSMA -prostate-specific membrane antigen, image-guided drug delivery, targeted therapy, theranostics, Aurora A kinase inhibition, MLN8237, antibody-theranostic conjugates

## Abstract

Prostate cancer (PC) is an aggressive cancer that can progress rapidly and eventually become castrate-resistant prostate cancer (CRPC). Stage IV metastatic castrate-resistant prostate cancer (mCRPC) is an incurable late-stage cancer type with a low 5-year overall survival rate. Targeted therapeutics such as antibody-drug conjugates (ADCs) based on high-affinity monoclonal antibodies and potent drugs conjugated via smart linkers are being developed for PC management. Conjugating further with *in vitro* or *in vivo* imaging agents, ADCs can be used as antibody-theranostic conjugates (ATCs) for diagnostic and image-guided drug delivery. In this study, we have developed a novel ATC for PSMA (+) PC therapy utilizing (a) anti-PSMA 5D3 mAb, (b) Aurora A kinase inhibitor, MLN8237, and (c) for the first time using tetrazine (Tz) and *trans*-cyclooctene (TCO) click chemistry-based conjugation linker (CC linker) in ADC development. The resulting 5D3(CC-MLN8237)_3.2_ was labeled with suitable fluorophores for *in vitro* and *in vivo* imaging. The products were characterized by SDS-PAGE, MALDI-TOF, and DLS and evaluated *in vitro* by optical imaging, flow cytometry, and WST-8 assay for cytotoxicity in PSMA (+/−) cells. Therapeutic efficacy was determined in human PC xenograft mouse models following a designed treatment schedule. After the treatment study animals were euthanized, and toxicological studies, complete blood count (CBC), blood clinical chemistry analysis, and H&E staining of vital organs were conducted to determine side effects and systemic toxicities. The IC_50_ values of 5D3(CC-MLN8237)_3.2_-AF488 in PSMA (+) PC3-PIP and PMSA (−) PC3-Flu cells are 8.17 nM and 161.9 nM, respectively. Pure MLN8237 shows 736.9 nM and 873.4 nM IC_50_ values for PC3-PIP and PC3-Flu cells, respectively. *In vivo* study in human xenograft mouse models confirmed high therapeutic efficacy of 5D3(CC-MLN8237)_3.2_-CF750 with significant control of PSMA (+) tumor growth with minimal systemic toxicity in the treated group compared to PSMA (−) treated and untreated groups. Approximately 70% of PSMA (+) PC3-PIP tumors did not exceed the threshold of the tumor size in the surrogate Kaplan-Meyer analysis. The novel ATC successfully controlled the growth of PSMA (+) tumors in preclinical settings with minimal systemic toxicities. The therapeutic efficacy and favorable safety profile of novel 5D3(CC-MLN8237)_3.2_ ATC demonstrates their potential use as a theranostic against aggressive PC.

## Graphical Abstract

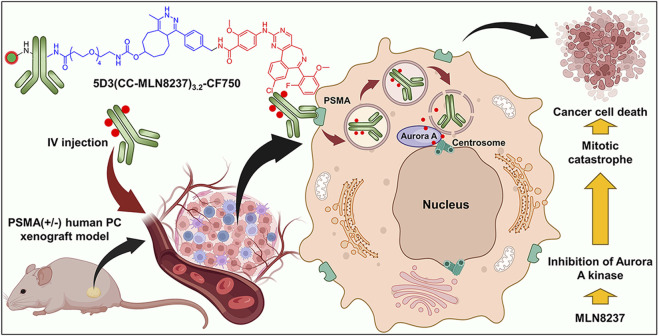

## 1 Introduction

Prostate cancer (PC) is estimated to account for almost 29% of all diagnosed cancer cases and 12% of cancer deaths in men in the United States (Siegel et al., 2023; Siegel et al., 2024). Radical prostatectomy, chemotherapy, radiotherapy, immunotherapy, and androgen/hormone deprivation therapy (ADT) are the standard of care at different stages of PC disease (Lindner et al., 2010; Lorente et al., 2016; Perera et al., 2016; Mottet et al., 2017; Evans, 2018). However, almost all PCs eventually become castrate-resistant prostate cancer (CRPC), which can progress rapidly and metastasize to stage IV metastatic castrate-resistant prostate cancer (mCRPC) (Sartor and de Bono, 2018; Einstein et al., 2021). Despite the continued progress in the treatment landscape of mCRPC in the last few decades from palliative care to taxane chemotherapy (e.g., docetaxel, cabazitaxel), sipuleucel-T immunotherapy, bone-specific radionuclide radium-223, androgen receptor directed therapies (e.g., abiraterone, enzalutamide), and poly (ADP-ribose) polymerase inhibitors, presently, there are no curative treatments for mCRPC (Antonarakis et al., 2020). Moreover, these existing therapies may be associated with poor tolerability and toxicity to healthy cells (Antonarakis et al., 2020). An increasing volume of clinical data proves the limitation of chemotherapeutics due to the lack of target-specificity, lack of efficacy in metastatic PC therapy, and induction of severe systemic toxicities in patients (Diamantis and Banerji, 2016; Rosenfeld et al., 2020; Vazquez et al., 2021; Wang et al., 2022). The off-targeted accumulation of these cytotoxic agents in healthy organs and tissues results in severe systemic toxicities and adverse side effects in PC patients. To overcome these issues, the development of image-guided and targeted drug delivery systems that enable early diagnosis of the disease and treatment are critically important to treat this disease effectively (Hapuarachchige and Artemov, 2020).

Image guidance is an essential approach in the early development of novel therapeutics and drug delivery systems. In the preclinical settings, imaging is used for several purposes including the non-invasive determination of pharmacokinetics, biodistribution, tumor uptake, off-target accumulation, controlled-release, and treatment response (Ojha et al., 2015). The use of image-guided drug delivery in clinics tremendously supports diagnostic imaging to recognize the targeted biomarkers, determine the position, size, and stage of cancer, and design the treatment plan in personalized medicine (Arranz and Ripoll, 2015; Hapuarachchige and Artemov, 2020). Hence, image-guided drug delivery systems are called theranostics when the delivery platform provides both diagnostic information and therapy (Herrmann et al., 2017).

Developing antibody-drug conjugates (ADCs) is a continuously thriving field for targeted drug delivery. An ADC consists of a target-specific antibody and cytotoxic agents conjugated through a linker. (Drago et al., 2021). Their target-specificity enhances therapeutic efficacy with minimal exposure to healthy tissue, expanding the therapeutic window of ADCs (Marei et al., 2022). Developing antibodies with high specificity and binding affinity on targeted cells, designing novel linkers for targeted-specific controlled release, and novel potent chemotherapeutics that can specifically kill the cancer cells vastly support the development of new ADCs for cancer therapy (Bakhtiar, 2016). Currently, following PC-specific antigens, (a) prostate-specific membrane antigen, PSMA, (b) trophoblast cell surface antigen-2, TROP-2, (c) six-transmembrane epithelial antigen of prostate-1, STEAP-1, (d) tissue factor, TF, (e) delta-like protein 3, DLL-3, (f) B7-H3 family of proteins, B7-H3, and (g) human epidermal growth factor receptor 2, HER2 are being studied as targets for the development of ADCs for PC therapy (Rosellini et al., 2021; Sardinha et al., 2023). Among these biomarkers, PSMA is one of the most well-known and clinically validated biomarkers that is expressed 1000-fold more on PC tumors than normal prostatic tissue (Ghosh and Heston, 2004; Jeitner et al., 2022). PSMA is internalized via clathrin-coated pits and subsequent endocytosis (clathrin-mediated endocytosis) upon ligand binding. Targeting PSMA is expected to result in high *in vitro* and *in vivo* image quality and cellular uptake by tumor cells due to these internalization characteristics that lead to enhanced tumor uptake and retention (Jeitner et al., 2022). Moreover, its overexpression in more than 70% of patients with mCRPC, in addition to its extracellular domain that can be targeted by antibodies and a motif that results in the internalization of bound agents, is what makes PSMA an attractive biomarker for diagnostic and therapeutic targeting (Donin and Reiter, 2018).

Recently, two PSMA-targeted ADCs (MLN2704 and PSMA-MMAE) have been used in clinical trials (Milowsky et al., 2016; Petrylak et al., 2020; Mjaess et al., 2023). However, both drugs were discontinued as they didn’t meet their primary endpoint due to the narrow therapeutic window, neurotoxicity effects, treatment-related adverse events, and lack of efficacy and safety in a significant percentage of patients (Galsky et al., 2008; Milowsky et al., 2016; Petrylak et al., 2020). The reported trial results of ADCs in PCs are indicative of the need for the development of novel, effective, and high-safety profile theranostics to treat advanced PCs. As of 2023, seven ADCs for PC are in phase I/II ongoing clinical trials (Sardinha et al., 2023). We have recently developed an anti-PSMA 5D3 monoclonal antibody, which can specifically bind to the extracellular domain of PSMA with sub-nanomolar affinity, and successfully used it as a therapeutic platform (Nováková et al., 2017; Huang et al., 2020). The unique characteristics of anti-PSMA 5D3 mAb include high target-specificity, enhanced binding affinity, fast internalization, and localization at the centrosome.

**FIGURE 1:**
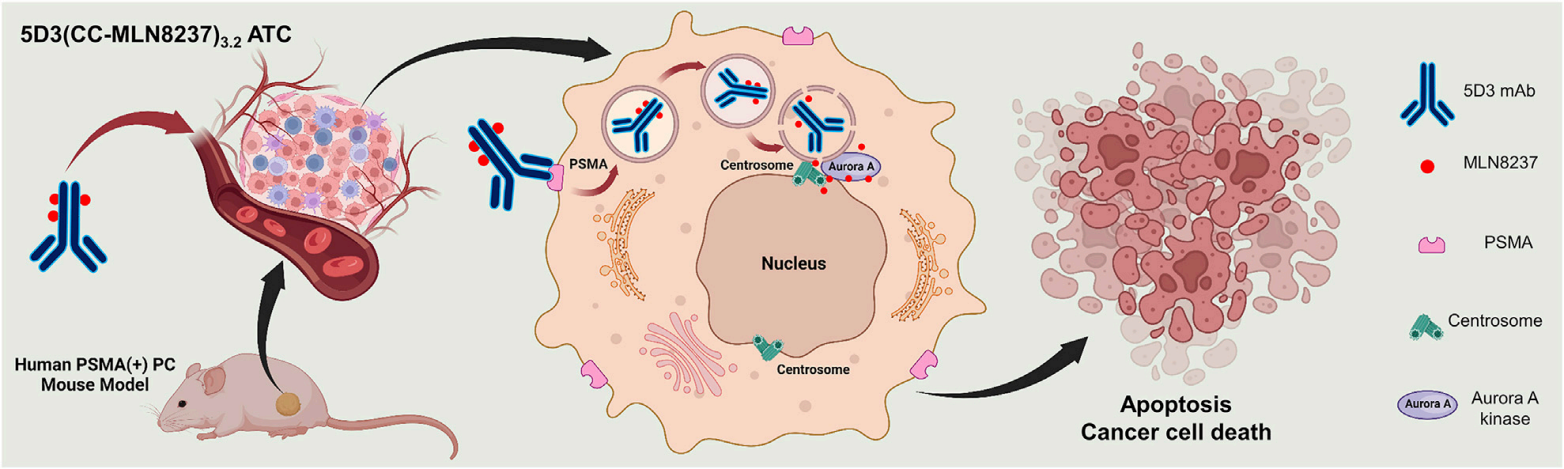
Schematic representation of the therapeutic approach using 5D3(CC-MLN8237)_3.2_ ATC in a PSMA (+) prostate cancer cell.

Aurora A kinase is a key mitotic regulator in the assembly of spindles, maturation of centrosomes, chromosomal segregation, and cytokinesis (Barr and Gergely, 2007; Görgün et al., 2010; Venkatakrishnan et al., 2015; Yan et al., 2016). The deregulation of Aurora A kinase is shown to induce mitotic arrest followed by apoptosis as well as severe mitotic abnormalities that result in selective lethality for many types of solid and hematological malignancies (Du and Hannon, 2004; Harrington et al., 2004; Cheung et al., 2009; Perez Fidalgo et al., 2009; Moore et al., 2010; Malumbres and Perez de Castro, 2014). Because of its significant role in mitotic progression and tumor proliferation (Cheung et al., 2009), Aurora A kinase inhibition would be expected to have an antitumor effect across a broad range of human tumors (Zhou et al., 2022). Alisertib (MLN8237) is a selective small molecular Aurora-A kinase inhibitor that is investigated for treatment as a single agent or in combination with other agents across a range of solid and hematologic malignancies (Manfredi et al., 2011; Venkatakrishnan et al., 2015; Tayyar et al., 2017). The high antitumor activity of MLN8237 through disruption of mitotic progression (Hoar et al., 2007; Nováková et al., 2017) can provide significant advantages as a drug for the development of the anti-PSMA 5D3-based ADC that targets PSMA (+) PC cells. In this study, we used Aurora A kinase inhibitor, MLN8237, for the first time as a conjugated chemotherapeutic using a novel click chemistry-based conjugation chemistry (CC linker) in ADC development with anti-PSMA 5D3 mAb. The resulting 5D3(CC-MLN8237)_3.2_ were labeled with imaging agents for image-guided evaluation of 5D3(CC-MLN8237)_3.2_ antibody-theranostic conjugates (ATCs). 5D3(CC-MLN8237)_3.2_ ATCs were fully characterized and evaluated *in vitro* for cytotoxicity and cellular uptake in PSMA (+/−) cells. *In vivo,* therapeutic efficacy was determined in bilateral/dual tumor human PC subcutaneous xenograft mouse models (Figure 1). At the end of the therapeutic study, animals were euthanized and evaluated for systemic toxicities by CBC study, clinical chemistry analyses of blood, and hematoxylin and eosin (H&E) staining.

## 2 Methods and materials

### 2.1 Cell lines

We used PSMA (+) PC3-PIP and PSMA (−) PC3-Flu PC cells in this study. Cells were kindly provided by Dr. Martin Pomper’s lab in the Johns Hopkins University School of Medicine. Cells were grown in RPMI 1640 medium supplemented with 10% FBS and 1% penicillin−streptomycin. In addition, the medium for PC3-PIP was supplemented with puromycin (20 μg/mL). Cells were maintained at 37°C in a humidified incubator with a 5% CO_2_ atmosphere. Cells were tested for *mycoplasma* and confirmed to be free of contamination. These cells were used for *in vitro* imaging, cytotoxicity studies, flow cytometric analysis, and for the preparation of mouse models for *in vivo* imaging, therapeutic studies, and toxicological analyses.

### 2.2 Antibody, chemicals, and solvents

5D3 mAb was produced following the protocol as previously described and stored in 0.02% NaN_3_/phosphate-buffered saline (PBS) for long-term storage (Nováková et al., 2017). The NaN_3_ was removed by buffer exchange using 30 kDa MWCO ultrafiltration centrifugal filter units purchased from Sigma-Aldrich, Inc. before use. Methyltetrazine amine.HCl and *trans*-cyclooctene PEG_4_-NHS (TCO-PEG_4_-NHS) were purchased from Click Chemistry Tools, Scottsdale, AZ, United States. The Aurora A kinase inhibitor drug, MLN8237 (Alisertib), was purchased from MCE (MedChemExpress) LLC, NJ, United States NHS esters of fluorophores, AlexaFluor 488, AlexaFluor 555, and CF750 dyes were purchased from MilliporeSigma Inc. and Biotium Inc., United States, respectively. Organic solvents and HPLC-grade water were purchased from Sigma-Aldrich and FisherScientific, Inc., respectively. Dulbecco’s phosphate-buffered saline (DPBS), and BupH phosphate-buffered-saline were purchased from Thermo Fisher, Inc. All chemicals, reagents, and solvents were used without further purification unless otherwise stated.

### 2.3 Synthesis of compounds

#### 2.3.1 Synthesis of MLN8237-Tz

**FIGURE 2:**
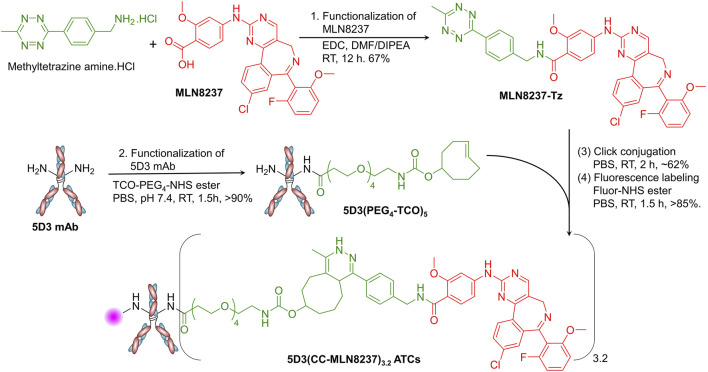
Synthesis of the 5D3(CC-MLN8237)_3.2_ ATCs. In the synthesis of 5D3(CC-MLN8237)_3.2_ ATC, first, (1) MLN8237 was functionalized with methyltetrazine using EDC-activated conjugation chemistry in DMF/DIPEA (Yield: 67%). (2) 5D3 mAb was functionalized with pegylated TCO using TCO-PEG_4_-NHS ester in PBS (Yield: >90%). (3) 5D3(PEG_4_-TCO)_5_ and MLN8237-Tz were conjugated by catalyst-free strain-promoted click reaction in PBS (Yield: ∼62%). (4) The product, 5D3(CC-MLN8237)_3.2_, was subsequently labeled with fluorophores for *in vitro* and *in vivo* imaging using NHS ester of fluorophores in PBS (Yield: >85%).

The syntheses were designed and conducted as shown in the Figure 2. MLN8237 (25 mg, 48.2 µmol), 1.2 equivalent of methytetrazine amine.HCl (13.7 mg, 57.8 µmoles), and 1.2 equivalent of 1-ethyl-3-(3-dimethylaminopropyl)carbodiimide (EDC, 9.0 mg, 57.8 µmoles) were taken into a micro-scale reaction vial and dissolved in dimethylformamide (DMF, 2.0 mL). The reaction mixture was treated with 1.0 µL of *N,N*-diisopropylethylamine (DIPEA) and stirred at room temperature for 12 h. The organic layer was evaporated using a rotavapor, and the crude solid product was purified by the HPLC-PDI system (Shimadzu LC-AD HPLC-PDI) equipped with a C18 reverse-phase column using a 10%–90% gradient acetonitrile/water with 0.1% Trifluoroacetic acid (TFA) mobile phase (Supplementary Figure S1). The desired pure product MLN8237-Tz was isolated as a pink-red solid (20 mg, yield 67%). The structure was confirmed by ^1^H NMR (Supplementary Figure S2, Bruker Avance III 500 MHz NMR spectrometer) and HPLC-MS (Agilent Infinity Lab LC/MSD XT system) equipped with a C18 reverse-phase column and a high-speed, sensitive single quadrupole mass spectrometer (Supplementary Figure S3). ^1^H NMR (500 MHz, CDCl3): δ 8.60 (s, 1H), 8.56 (d, J = 8 Hz, 2H), 8.32 (m, 2H), 8.25 (d, J = 9 Hz, 2H), 7.79 (bs, 1H), 7.73 (d, J = 10 Hz, 2H), 7.58 (d, J = 5 Hz, 2H), 7.44 (m, 1H), 7.40 (bs, 1H), 6.77 (bs, 2H), 4.80 (s, 2H), 3.99 (s, 3H), 3.08 (s, 3H), 1.24 (s, 3H). HPLC-MS (m/z): [M+H]^+^ calcd for C_38_H_29_ClFN_9_O_3_ 702.2, found 702.3.

#### 2.3.2 Synthesis of 5D3(PEG_4_-TCO)_5_

5D3 mAb (20 mg, 0.133 µmol) in PBS (1.0 mL) was treated with 10 equivalent of TCO-PEG_4_-NHS ester (0.35 mg, 1.33 µmol in 10 µL of anhydrous DMSO) and stirred for 1.5 h at room temperature (Figure 2). Unreacted reagents and byproducts were removed by ultrafiltration using 10 kDa MWCO (4 mL) centrifugal filter units, and the product was further purified by size exclusion column (SEC) chromatography using an ÄKTA go protein purification system (Cytiva Life Sciences, MA, United States). The pure product, 5D3(PEG_4_-TCO)_5_ was isolated with over 90% yield and it was characterized by sodium dodecyl sulfate-polyacrylamide gel electrophoresis (SDS-PAGE), matrix assisted desorption/ionization-time of flight (MALDI-TOF), and dynamic light scattering (DLS).

#### 2.3.3 Synthesis of 5D3(CC-MLN8237)_3.2_ ATCs

**FIGURE 3:**
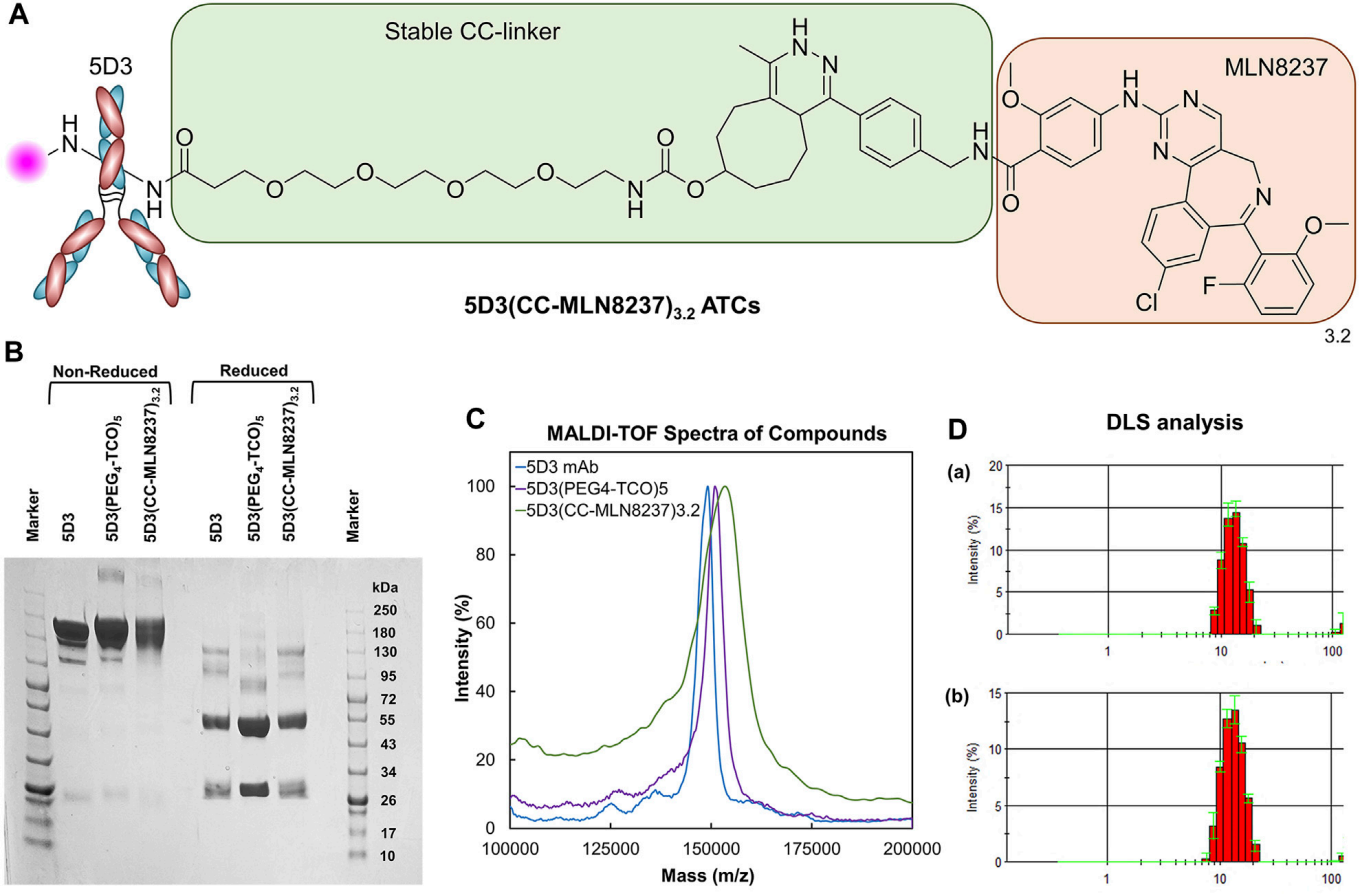
Structure and Characterization of 5D3(CC-MLN8237)_3.2_. **(A)** The structure of 5D3(CC-MLN8237)_3.2_ ATCs. **(B)** SDS-PAGE analysis of 5D3 mAb, intermediate 5D3(PEG_4_-TCO)_5_, and 5D3(CC-MLN8237)_3.2_ under non-reducing and reducing conditions. **(C)** MALDI-TOF spectra of 5D3 mAb, intermediate 5D3(PEG_4_-TCO)_5_, and 5D3(CC-MLN8237)_3.2_. **(D)** Dynamic light scattering analysis (DLS) and size distribution of **(** **a** **)** 5D3 mAb (12.7 nm) and **(** **b** **)** 5D3(CC-MLN8237)_3.2_ (13.4 nm).

5D3(PEG_4_-TCO)_5_ (10.0 mg, 66.67 nmoles in 1.0 mL of PBS) was taken into a 1.5 mL Eppendorf tube and treated with MLN8237-Tz (2.34 mg, 33.33 µmole in 20 µL of anhydrous DMSO). The mixture was vortexed and stirred gently using a tube rotator for 3 h at room temperature. Unreacted reagents and byproducts were removed by ultrafiltration using 10 kDa MWCO (4 mL) centrifugal filter units, and the product was further purified by SEC chromatography to obtain 5D3(CC-MLN8237)_3.2_ with approximately 62% yield. For fluorescent labeling, the product (2–5 mg/mL) was treated with NHS-ester form of Alexa Fluor 488, Alexa Fluor 555, or CF750 (5 mol equiv. in 10 µL of anhydrous DMSO) and stirred at room temperature in PBS for 1.5 h. The reaction mixture was purified by ultrafiltration (10 kDa MWCO) followed by SEC chromatography as described earlier to obtain pure final products, fluorescent 5D3(CC-MLN8237)_3.2_ ATCs (Figure 3A) with over 85% yield. The degree of fluorescence labeling (DOL) was calculated following the manufacturer’s protocol (DOL = 1–2). All ATC samples were stored in 1X PBS at 4°C.

### 2.4 Characterization of compounds

#### 2.4.1 SDS-PAGE study

Unconjugated 5D3 mAb, 5D3(PEG_4_-TCO)_5_, and 5D3(CC-MLN8237)_3.2_ were analyzed by SDS-PAGE under reducing and non-reducing conditions following standard lab procedures (Figure 3B). A 4%–20% Mini-Protean TGX gel (Bio-Rad Laboratories Inc., United States) was used, and a color protein standard broad-range (10–250 kDa) molecular weight marker (New England Biolabs Inc., MA, United States) was co-run to estimate molecular weights of proteins. Protein samples (3–4 μg/mL) were heated for 10 min at 70°C in NuPAGE sample reducing agent and non-reducing sample buffer (ThermoFisher Scientific, MA, United States). Samples were subsequently loaded into the wells in a volume of 10 μL and ran on Mini-PROTEAN Tetra Vertical Electrophoresis Cell (Bio-Rad Laboratories Inc., United States). Gels were stained with Coomassie Brilliant Blue R-250 Staining Solution (Bio-Rad laboratories, United States) and imaged using the Chemidoc Touch Gel Imaging System.

#### 2.4.2 MALDI-TOF study

For the MALDI-TOF analysis, a 1.0 µL sample (5 mg/mL in H_2_O) was spotted on a targeted plate, and 1.0 µL of sinapinic acid (10 mg/mL in 1:1 H_2_O:CH_3_CN, 0.1% TFA) was co-spotted after drying in air. MALDI-TOF spectra were acquired after the spot was completely dry in the air on a Voyager DE-STR MALDI-TOF mass spectrometer (Mass Spectrometry and Proteomics Facility, JHU School of Medicine). The degree of conjugation (DOC) of PEG_4_-TCO and drug-to-antibody ratio (DAR) were calculated based on the change of molecular weights measured by MALDI-TOF (Figure 3C). The DOC for PEG_4_-TCO and the DAR were 5 and 3.2, respectively.

#### 2.4.3 Flow cytometry and DLS study

Cells grown to approximately 90% confluency were harvested by trypsinization. Twelve cell samples in 200 µL of media (0.2 million cells/sample) were treated with 5D3-AF488 or 5D3(CC-MLN8237)_3.2_-AF488 (20 μg/mL) and incubated at 37°C for 1.5 h. Unbound mAb and ATCs were washed using DPBS and the cells were then fixed by 4% paraformaldehyde (PFA). Isolated cell samples were filtered and analyzed by SONY SH800 flow cytometer (Supplementary Figure S4). Data was analyzed and fluorescence histograms were generated by FlowJo software (BD Biosciences, United States). The hydrodynamic diameter of free 5D3 mAb and 5D3(CC-MLN8237)_3.2_ (1.0 mg/mL) was determined by DLS (Figure 3D) using a Nano-ZS90 Zetasizer (Malvern Instruments, UK).

### 2.5 *In vitro* fluorescence imaging study

PSMA (+) PC3-PIP or PSMA (−) PC3-Flu cells (0.2×10^6^ cells/chamber in 500 µL of media) were seeded in 4-well chamber slides (Nunc Lab-Tek, Thermo Scientific) and grown to ∼90% confluency (24–48 h). Cells were then fed with fresh media containing 5D3-AF555 or 5D3(CC-MLN8237)_3.2_-AF555 (20 μg/mL) and incubated at 37°C for 1.5 h. Cells were washed twice with 1X DPBS and fixed using 4% PFA in PBS on ice for 10 min. After washing once with DPBS, cells were permeabilized with 0.1% Triton X-100 for 5 min and then blocked with 1.0% BSA/10% goat serum/0.3 M glycine in 0.1% PBS-Tween for 1 h. After a quick wash with 1X DPBS, cells were treated with centrosome marker, recombinant anti-pericentrin Alexa Fluor^®^ 488 antibody (Abcam, Inc, 1:500 dilution), and anti-α-tubulin Alexa Fluor^®^ 647 (Abcam, Inc. 1:250 dilution) for overnight at 4°C. Cell nuclei were counterstained with Hoechst 33342 (10 μg/mL in H_2_O at room temperature for 10 min). After washing with 1X DPBS twice, the slides with cells were wet mounted. Cells were imaged using a Zeiss LSM880-Airyscan FAST super-resolution single-point, laser scanning confocal microscope. Images were processed using Zeiss Zen software.

### 2.6 Determination of cytotoxicity

The cytotoxicity of 5D3(CC-MLN8237)_3.2_-AF488 was determined by treating PC3-PIP and PC3-Flu (control) cells in 96-well plates in RPMI 1640 culture media in the presence of 5D3-(CC-MLN8237)_3.2_-AF488 over a series of concentrations. Cells were seeded in a 96-well plate (2000 cells/well) and grown for 24 h to achieve 30%–40% confluency. Then, cells were treated with increasing concentrations of 5D3(CC-MLN8237)_3.2_-AF488 or pure MLN8237 equivalent to the drug concentration in 5D3-(CC-MLN8237)_3.2_-AF555 (1.0 ng/mL-1.0 mg/mL by 10-fold increment). After the incubation for 72 h, IC_50_ values of 5D3(CC-MLN8237)_3.2_-AF488 ATC and corresponding free MLN8237 were determined using the WST-8 assay (CCK-8 assay, Dojindo Molecular Technologies), following the manufacturer’s protocol. Briefly, media in each well were replaced by 100 μL of the fresh media. Then, cells in each well were treated with 10 μL of WST-8 reagent and incubated at 37°C for 3 h. During incubation, WST-8 tetrazolium salt is reduced by dehydrogenase in living cells, forming a yellow formazan dye with λ_max_ at 450 nm. After 3 h, the absorbance in wells was measured at 450 nm using a BioTek Epoch microplate spectrophotometer (Agilent Technologies). The concentration of the formazan dye in the media produced by dehydrogenases is directly proportional to the density of viable cells per well. The cell viability of treated cells was normalized to readings in untreated control cells with 100% viability. Data were fitted, and IC_50_ values for free MLN8237 and 5D3(CC-MLN8237)_3.2_-AF488 were calculated using Prism 9 software (GraphPad, San Diego CA).

### 2.7 Human PC xenograft animal models

Healthy, four-to-six weeks-old, athymic, nude male mice were purchased from Charles River Laboratories (United States) and maintained in the animal facility in Miller Research Building (B-level) of the Johns Hopkins School of Medicine (at 25°C/12 h light-dark cycle/food and water *ad libitum*). The bilateral PSMA (+/−) dual-tumor subcutaneous xenograft mouse models were prepared for the *in vivo* experiments. Mice were inoculated on the right and left flanks with PC3-PIP and PC3-Flu cells (2 × 10^6^ cells in 50 μL of 1:1 RPMI 1640/Matrigel). The treatment started when tumor size was approximately 0.5 cm, a volume of approximately 65 mm^3^ (after 7–10 days). Tumor volumes were calculated using the formula (L×W^2^)π/6, where L is the longest diameter (the major axis), and W is the tumor width, measured perpendicular to the major axis.

All animal experiments were conducted strictly following the animal protocol approved by the Animal Care and Use Committee (ACUC) at the Johns Hopkins University School of Medicine.

We measured the tumor sizes using a digital caliper every other day to make sure not to exceed the tumor sizes subcutaneous xenograft tumor size above 2 cm in any direction. Animals were euthanized immediately when the tumor size exceeded the 2 cm limit or the tumor volume reached the 2000 mm^3^ limit following the approved protocol. All mice were sacrificed at the end of the treatment period by 3% isoflurane-assisted cervical dislocation.

### 2.8 *In vivo* and *ex vivo* optical imaging and therapeutic study

Mice were randomly selected into treatment and control groups (*n* = 10). The first dose of 5D3(CC-MLN8237)_3.2_-CF750 (120 µL of 5.0 mg/kg in PBS) was injected through the tail vein. *In vivo* optical images were taken using Xenogen IVIS *in vivo* small animal imaging system using 745 nm as excitation wavelength (λ_ex_) and collecting the fluorescence signals at emission wavelength (λ_emi_) 820 nm on day 4. Tumor sizes were measured every other day during the treatment period using a digital caliper and tumor volumes were calculated using the formula (L×W^2^)π/6. The second dose was administered on day 14 of the treatment period and measuring tumor sizes was continued for up to 21 days. Relative tumor volume (*V*_*t*_ */V*_*0*_) was calculated and plotted against time (days) based on the tumor volume at day t (*V*_*t*_) with respect to the tumor volume at day 0. We have also conducted a biodistribution study by *ex vivo* optical imaging of tumor tissues and selected vital organs extracted from treated mice after 96 h of drug administration.

### 2.9 Toxicological study

Daily clinical observations and body weight measurements were conducted every other day as a part of a toxicological assessment during the treatment period. After the 21-days treatment period, we investigated possible *in vivo* toxicities of 5D3(CC-MLN8237)_3.2_-CF750 by (a) determining the complete blood count (CBC) and (b) analyzing the blood clinical chemistry profiles related to liver and kidney toxicities. Blood collections by cardiocentesis were performed at the time of euthanasia of mice in both treatment and control groups under sterile conditions in a biosafety cabinet. The total serum protein (T-Pro), blood urea nitrogen (BUN), alkaline phosphatase (ALP), creatinine (Cre), and alanine transaminase (ALT) levels were measured using Spotchem EZ vet veterinary chemistry analyzer (Scil Animal Care Company, Gurnee, Illinois, United States). Red blood cells (RBC), white blood cells (WBC) and platelet (PLT) contents were counted using the scil Vet abc Plus + hematology analyzer (Scil Animal Care Company, Gurnee, Illinois, United States). All aqueous solutions were prepared with ultrapure water. Following blood collections, tissues of tumors and vital organs, brain, lungs, heart, liver, kidneys, and spleen, were extracted and stained by H&E for pathological evaluation. Tissue samples were preserved in 10% neutral buffered formalin, later processed, and embedded in paraffin. H&E staining was performed on 4 μm sections following a standard protocol by the Reference Histology Laboratory, Department of Pathology, The Johns Hopkins Hospital.

### 2.10 Statistical analysis

The cell viability study was performed in triplicate independent experiments with triplicates per plate for statistical analyses. The one-way analysis of variance (ANOVA) was used for the omnibus F-test. In *in vivo* experiments, the statistical analysis (*t*-test) between treated and untreated groups was performed using JMP 12.1.0 Statistical Discovery from SAS. A *p*-value of less than 0.05 was considered significant (*p < 0.05). Survival curves in Kaplan−Meier analysis use the time when the tumor has reached over 2000 mm^3^ increase in the volume relative to the initial tumor volume as the surrogate endpoint to develop the Kaplan−Meier graph.

## 3 Results and discussion

### 3.1 Synthesis of 5D3(CC-MLN8237)_3.2_ ATC and CC linker chemistry

5D3(CC-MLN8237)_3.2_ ATCs are composed of an anti-PSMA 5D3 mAb conjugated with Aurora A kinase inhibitor (MLN8237) via a non-cleavable TCO-Tz-based click linker (Figure 2). This click linker is stable in the circulatory system because the drug was conjugated through an amide/peptide bond to the linker. Proteolysis of amide/peptide bonds is naturally catalyzed by intracellular enzymes predominantly in lysosomes (Yang et al., 2023). The concentration of proteolytic enzymes in the circulatory system is extremely low. Hence, the MLN8237 conjugated by the amide bond is stable in the circulatory system. The TCO- and Tz-functionalized 5D3(PEG_4_-TCO)_5_ and MLN8237-Tz undergo rapid, catalyst-free click chemistry. This reaction occurs with a kinetic rate ranging from 2 × 10^3^ to 1 × 10^6^ M^−1^s^−1^ under physiological conditions, driven by inverse electron demand Diels–Alder (IEDDA) conjugation chemistry (Blackman et al., 2008). Remarkably, this process produces only non-toxic N_2_ as a byproduct, without any cross-reactions with other functional groups. Additionally, pegylation of the linker enhances water solubility and biocompatibility of the resulting conjugate (Veronese and Mero, 2008). These advantages position our approach favorably compared to the common uncleavable heterobifunctional MCC linker chemistry for antibody-drug conjugation. 5D3(CC-MLN8237)_3.2_ was labeled with suitable fluorophores for *in vitro* or *in vivo* imaging. We conjugated only 1-2 fluorophores per antibody. Based on our previous studies and experience, these small molecular fluorophores (Molecular Weight <1.0 kDa) have not made any contributions or interference to cytotoxicity, therapeutic efficacy, internalization, or tumor uptake of the drug delivery system.

### 3.2 SDS-PAGE analysis

The SDS-PAGE analysis was conducted for 5D3(CC-MLN8237)_3.2_ parallel with starting 5D3 mAb and activated 5D3(PEG_4_-TCO)_5_ under both non-reduced and reduced conditions (Figure 3B). We observed the high purity of activated 5D3(PEG_4_-TCO)_5_ and 5D3(CC-MLN8237)_3.2_, same as starting 5D3 mAb, and all biomolecules revealed expected molecular weight of 150 kDa in non-reduced conditions. The standard pattern of bands was also observed under reduced condition, showing a heavy chain band at ∼50 kDa and a light chain band at ∼25 kDa. 5D3(CC-MLN8237)_3.2_ had slightly decreased gel mobility relative to the unmodified 5D3 mAb; however, it is not pronounced since the conjugated drug, MLN8237 is a small molecule. Under reduced condition, the heavy chain had lower mobility, therefore, we suggest that the drug is conjugated to the heavy chain.

### 3.3 MALDI-TOF analysis

MALDI-TOF spectra of 5D3 mAb, 5D3(PEG_4_-TCO)_5_, and 5D3(CC-MLN8237)_3.2_ were obtained using sinapinic acid-based matrix (Figure 3C). The TCO functionalization (DOF) and DAR were calculated based on changes in the molecular weights after each conjugation step. Based on MALDI-TOF calculation, the average DOF of PEG_4_-TCO groups is 5, and DAR is 3.2 in 5D3(CC-MLN8237), which are optimal levels to maintain the solubility in aqueous media, binding affinity, and pharmacokinetics. Our previous studies showed that DAR less than four including hydrophobic drug conjugation does not significantly increase lipophilicity (logP <5) and often exhibits optimal solubility in aqueous media studies (Huang et al., 2020). As a proof-of-concept study, we conjugated 3.2 drugs per antibody and used direct amine conjugation chemistry to minimize the mAb exposure in reaction conditions at reduced temperatures.

### 3.4 Flow cytometry study and DLS characterization

The flow cytometry study was conducted to determine any significant changes in the binding affinity of 5D3 after drug conjugation and fluorescence labeling in PSMA (+) PC3-PIP cells and compared with PSMA (−) PC3-Flu cells. The results in Supplementary Figure S4 show no significant difference in binding affinity between 5D3 mAb (i) and 5D3(CC-MLN8237)_3.2_-AF488 (ii) in PSMA (+) cells. This result confirms the preservation of 5D3 immunoreactivity and specificity to PSMA (+) PC3-PIP cells upon drug conjugation and fluorescent labeling. There was no 5D3(CC-MLN8237)_3.2_-AF488 affinity to PSMA (−) PC3-Flu cells (Supplementary Figure S4 (iii)/(iv)). The hydrodynamic diameters of 5D3 mAb and 5D3(CC-MLN8237)_3.2_ were 12.7 and 13.4 nm, respectively. There is an insignificant increase in hydrodynamic diameter after conjugation (Figure 3D); however, it does not affect the binding affinity and internalization kinetics, as shown in the *in vitro* study results below.

### 3.5 5D3(CC-MLN8237)_3.2_-AF555 *in vitro* optical imaging study

**FIGURE 4:**
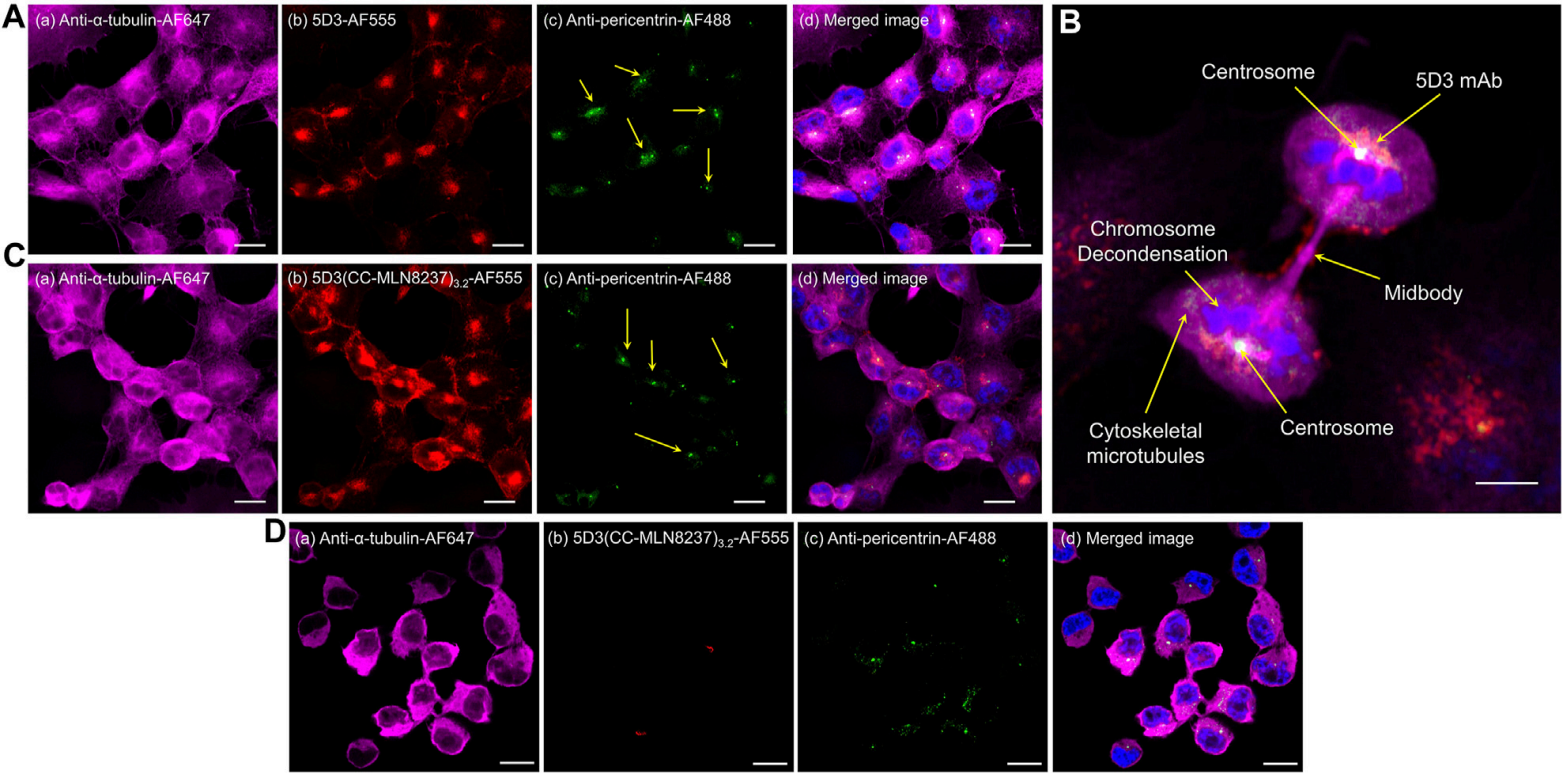
Super-resolution confocal fluorescence images of PSMA (+) PC3-PIP or PSMA (−) PC3-Flu cells. Panel **(A)** PSMA (+) cells treated with anti-α-tubulin-Alexa Fluor-647 **(** **a** **)**, 5D3-AF555 **(** **b** **)**, and anti-pericentrin Alexa Fluor-488 **(** **c** **)**, with channel merged image with the blue channel for Hoechst 33342 nuclei counterstaining **(** **d** **)**. Panel **(B)** Telophase of PSMA (+) mitotic cell showing the localization of 5D3 mAb at the centrosome. Cells treated with anti-α-tubulin-Alexa Fluor-647, 5D3(CC-MLN8237)_3.2_-AF555, anti-pericentrin Alexa Fluor-488, and Hoechst 33342 for nuclei counterstaining. Panel **(C)** PSMA (+) cells treated with anti-α-tubulin-Alexa Fluor-647 **(** **a** **)**, 5D3(CC-MLN8237)_3.2_-AF555 **(** **b** **)**, and anti-pericentrin Alexa Fluor-488 **(** **c** **)**, with channel merged image with the blue channel for Hoechst 33342 nuclei counterstaining **(** **d** **)**. Panel **(D)** PSMA (−) cells treated with anti-α-tubulin-Alexa Fluor-647 **(** **a** **)**, 5D3(CC-MLN8237)_3.2_-AF555 **(** **b** **)**, and anti-pericentrin Alexa Fluor-488 **(** **c** **)**, with channel merged image with the blue channel for Hoechst 33342 nuclei counterstaining **(** **d** **)**. (Scale bar: 10 μm, Magnification: ×63).

PSMA (+) PC3-PIP cells were labeled with fluorescent 5D3-AF555 and 5D3(CC-MLN8237)_3.2_-AF555 conjugates and incubated for internalization and localization in the subcellular components. Centrosomes of fixed cells were labeled with anti-pericentrin Alexa Fluor-488, a centrosome marker, and high-resolution confocal fluorescence images were acquired as shown in Figure 4. We observed the internalization of 5D3-AF555 and localization in the cytoplasm in PSMA (+) PC3-PIP cells (Figure 4A) and higher cellular uptake compared to PSMA (−) PC3-Flu cells (Supplementary Figure S5). Figure 4B and Supplementary Figure S6 show the super-resolution fluorescence image of a dividing PC3-PIP cell at telophase, visualizing the colocalization of 5D3-AF555 and anti-pericentrin-Alexa Fluor-488 marker, proving that 5D3 localizes in the proximity of centrosomes immediately after internalization. As shown in Figure 4C, 5D3(CC-MLN8237)_3.2_-AF555 also internalizes faster and localizes at the centrosomes, the same as free 5D3 mAb. Specific uptake of 5D3(CC-MLN8237)_3.2_-AF555 was not observed in PSMA (−) PC3-Flu cells (Figure 4D). We have previously quantitatively analyzed the cell surface binding and cellular uptake of 5D3 mAb, Fab fragments of 5D3 and their conjugates in PC3-PIP and PC3-Flu cells; we have not observed a significant change of internalization rate of 5D3 antibody upon drug conjugation (Hapuarachchige et al., 2020).

### 3.6 Determination of *in vitro* cytotoxicity of 5D3(CC-MLN8237)_3.2_-AF488

**FIGURE 5:**
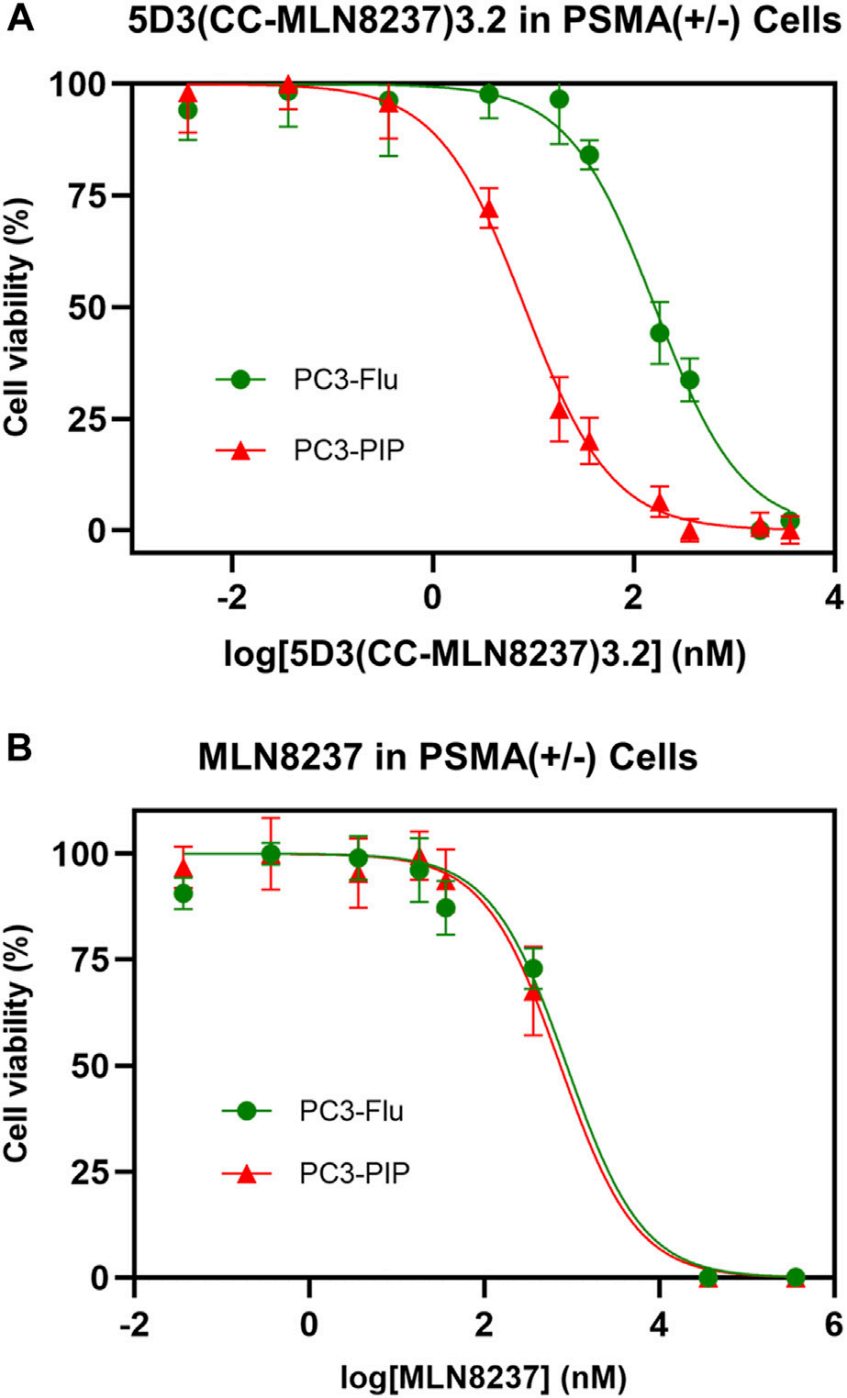
Effect of 5D3(CC-MLN8237)_3.2_-AF488 and free MLN8237 on PSMA (+/−) cell viability. **(A)**. Cytotoxicity of 5D3(CC-MLN8237)_3.2_-AF488 in PSMA (+) PC3-PIP cells (IC_50_ = 8.17 nM) and PSMA (−) PC3-Flu cells (IC_50_ = 161.9 nM). **(B)**. Cytotoxicity of free MLN8237 in PSMA (+) PC3-PIP cells (IC_50_ = 736.9 nM) and PSMA (−) PC3-Flu cells (IC_50_ = 873.4 nM). The cell viability was assessed with the WST-8 assay after 72 h of treatment with 5D3(CC-MLN8237)_3.2_-AF488 or equivalent free MLN8237 drug. The study was performed in triplicate per plate, and triplicate independent experiments, 5D3(CC-MLN8237)_3.2_-AF488 is 19.8 fold more efficacious in PSMA (+) cells than in PSMA (−) cells and 90.1 fold more efficacious than free MLN8237 drug in PSMA (+) cells. *p < 0.05.

The *in vitro* cytotoxicity of 5D3(CC-MLN8237)_3.2_-AF488 on PSMA (+) PC3-PIP cells was evaluated using PSMA (−) PC3-Flu cells and free MLN8237 drug as controls. The concentration of MLN8237 in 5D3(CC-MLN8237)_3.2_-AF488 and free MLN8237 was used to directly compare IC_50_ values (Figure 5). To directly compare cytotoxicity, the dose was normalized for MLN8237 concentrations in the nanomolar range. As shown in Figure 5A, we observed a significantly high cytotoxicity by 5D3(CC-MLN8237)_3.2_-AF488 in PSMA (+) PC3-PIP cells (IC_50_ = 8.17 nM) compared to the cytotoxicity in PSMA (−) PC3-Flu cells (IC_50_ = 161.9 nM). PC3-PIP and PC3-Flu cells treated with the equivalent concentration of free MLN8237 show IC_50_ values of 736.9 and 873.4 nM, respectively (Figure 5B). These results suggest that the cytotoxicity of 5D3(CC-MLN8237)_3.2_ was target-mediated by the anti-PSMA antibody.

### 3.7 *In vivo* and *ex vivo* imaging and determination of therapeutic efficacy

**FIGURE 6:**
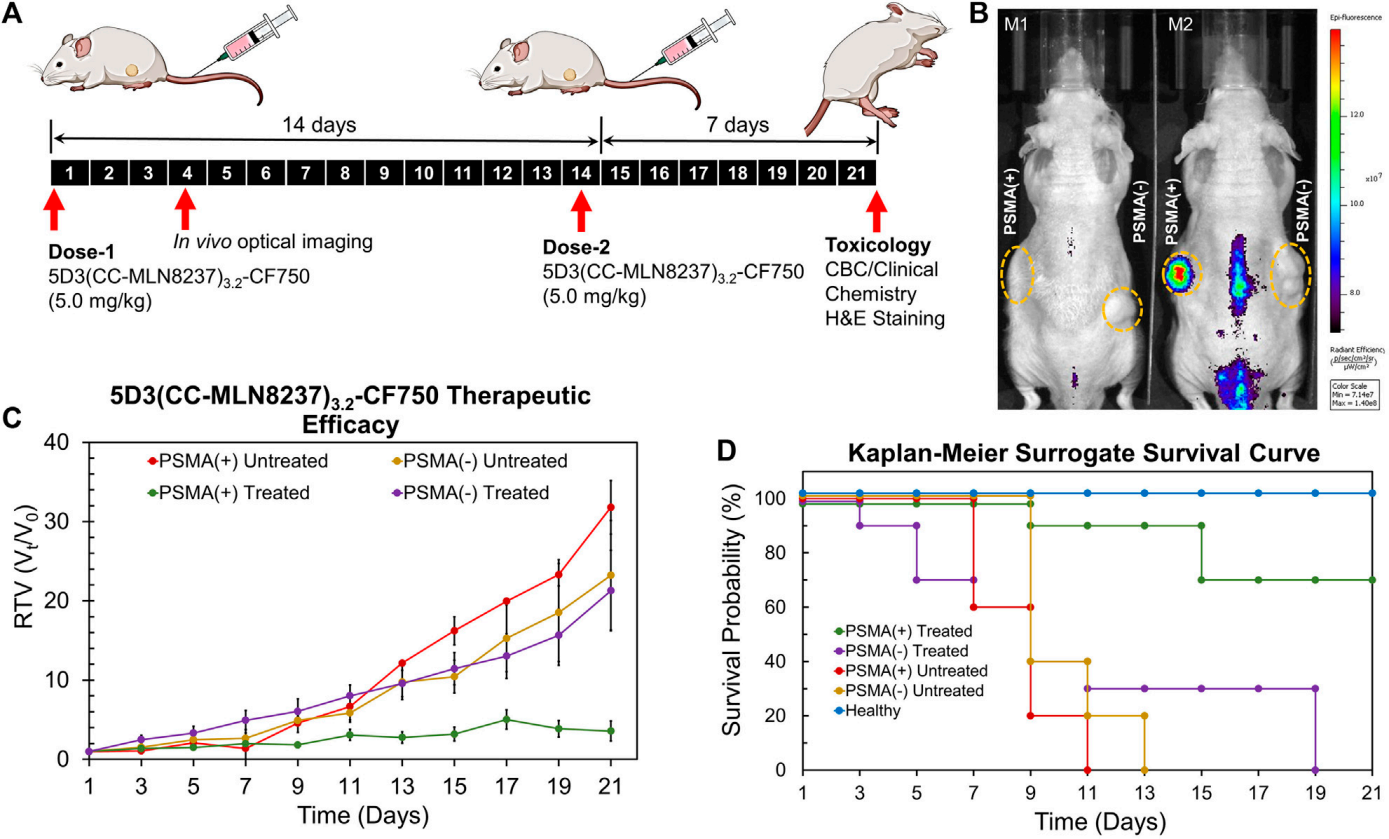
Imaging and therapeutic study in animal models. **(A)** Treatment schedules. Mice were inoculated with PSMA (+) PC3-PIP and PSMA (−) PC3-Flu cells. Two doses of 5D3(CC-MLN8237)_3.2_-CF750 (5.0 mg/kg) were given on day 1 and day 14 and tumor sizes were measured every other day for 21 days. **(B)** IVIS Xenogen images (λ_ex_:745 nm/λ_emi_: 820 nm) of tumor-bearing mice (M1: untreated mouse and M2: treated mouse) on day 4 showed high tumor uptake of 5D3(CC-MLN8237)_3.2_-CF750 by PSMA (+) tumor (left flank) in treated mouse. **(C)** Change of relative tumor volume (*V*_*t*_ */V*_*0*_) against the time of treatment period. The graph show the significant control of PSMA (+) tumor growth. **(D)** Kaplan-Meier surrogate survival curves showing higher overall survival of mice bearing PSMA (+) tumors and treated by 5D3(CC-MLN8237)_3.2_-CF750 (green, 70%) compared to PSMA (−) tumors in treated mice (purple, 0% on day 19), untreated mice with PSMA (+) tumors (red, 0% on day 11), and untreated mice with PSMA (−) tumors (yellow, 0% on day 13), and untreated healthy mice (blue, 100% during the treatment period). *n* = 10, *p < 0.05.

The *in vivo* therapeutic efficacy studies of 5D3(CC-MLN8237)_3.2_-CF750 were conducted in PSMA (+/−) bilateral/dual human PC subcutaneous tumor xenograft mouse models following the therapeutic schedule shown in Figure 6A. In this study, two groups of mice (n = 10) with both PSMA (+) and PSMA (−) tumors were treated with 5.0 mg/kg dose of 5D3(CC-MLN8237)_3.2_-CF750 on day 1. We have administered two doses (5.0 mg/kg per dose) on day 1 and day 14 in this proof-of-concept study based on our previous dose establishment of *in vivo* therapeutic studies of 5D3 mAb-ADCs. Tumor sizes were measured every other day by a caliper, and the second dose was administered on day 14. *In vivo* optical images were taken at day 1, 3, and 4 post-injection time points using a Xenogen IVIS small animal imaging system, shown in Figure 6B. A higher tumor uptake of 5D3(CC-MLN8237)_3.2_-CF750 in PSMA (+) tumors was exhibited, proving the tumor specificity of the ATC compared to PSMA (−) tumors. We observed signals in the lower abdominal area mainly due to the bladder, urine contamination, or autofluorescence from feces. PC3-PIP tumor uptake of 5D3(CC-MLN8237)_3.2_-CF750 is significantly higher than its uptake in PC3-Flu tumors as shown in the *ex vivo* biodistribution study. There was high liver uptake, which was not detected in whole body imaging due to the limitation of fluorescent signal penetration through the body tissues. The tumor measurement was continued for a total of 21 days. Remarkably, PSMA (+) tumor growth ceased during the 5D3(CC-MLN8237)_3.2_-CF750 treatment period (Figure 6C). In contrast, PSMA (+) and PMSA (−) tumors in untreated groups and PSMA (−) tumors in the treated group grew rapidly during the treatment period.

The Kaplan–Meier surrogate survival results for untreated mice and mice treated with 5D3(CC-MLN8237)_3.2_-CF750 are shown in Figure 6D. We used 2000 mm^3^ tumor size limit for the surrogate survival criterion. The results demonstrated that the 5.0 mg/kg treatment increased the survival of mice with PSMA (+) tumors (*p < 0.05), and 70% of PSMA (+) treated tumors did not reach the threshold level of tumor sizes during the treatment. In the treated group, 1 mouse died on day 9, and the tumors of the two mice became ulcerous and started to bleed. These two mice were euthanized according to the protocol on day 15. All mice in treated and untreated groups with PSMA (−) tumors and untreated PSMA (+) tumors exceeded the threshold tumor volume of 2000 mm^3^ before day 19.

Since Aurora A kinase is an intracellular enzyme, MLN8237 is effective upon intracellular drug release. MLN8237 is conjugated with the stable TCO-Tz linker via an α-peptide type amide bond. Amide bonds are hydrolysable by lysosomal enzymes such as proteases, amidases and cutinases (Rajagopalan and Kroutil, 2011; Mahesh et al., 2018) and it is also cleavable in acidic conditions (Dhavale et al., 2018). Therefore, we suggest that MLN8237 is released as free drug without traces of the linker by breaking this bond by the combination of enzymatic cleavage and acidic hydrolysis. We observed a significantly slower PSMA (+) tumor growth in treated mice (*p < 0.05) compared to the PSMA (−) tumors and PSMA (+) and PSMA (−) tumors in untreated mice from day 9 onward. PSMA (−) tumor growth was slower in both untreated and treated groups; however, this effect was insignificant. The overall efficacy of treatment was higher in PSMA (+) tumors and exhibited significantly (*p < 0.05) higher therapeutic efficacy in PSMA (+) tumors compared to PSMA (−) tumors.

### 3.8 Post-treatment evaluation of toxicological effects

**FIGURE 7:**
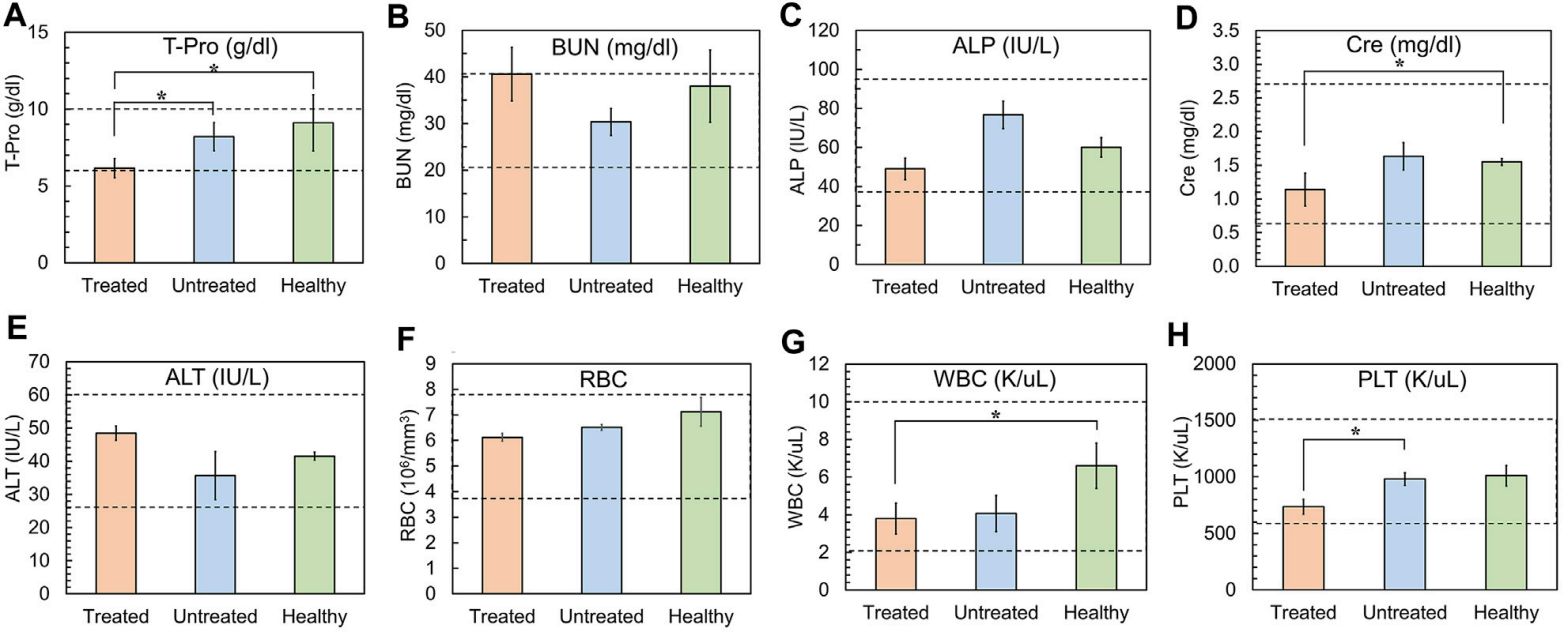
Analyses of clinical chemistry profile and, RBC, WBC, and PLT counts. **(A)** Total serum protein (T-Pro, normal range: 6.0–10.0 g/dL), **(B)** Blood Urea Nitrogen (BUN, normal range: 20–40 mg/dL), **(C)** Alkaline Phosphatase (ALP, normal range: 35–96 IU/L), **(D)** Creatinine (Cre, normal range: 0.60–2.72 mg/dL), **(E)** Alanine Transaminase (ALT, normal range: 25–60 U/L), **(F)** Red Blood cell Count (RBC, normal range: 3.8-7.9 ×10^6^/µL, **(G)** White Blood cell Count (WBC, normal range: 2–10 K/μL) **(H)** Platelet Count (PLT, normal range: 600–1500 K/μL). Gray area: normal range of analytes in healthy mice. (Plotted with standard error bars, *n* = 5, *p < 0.05).

**FIGURE 8:**
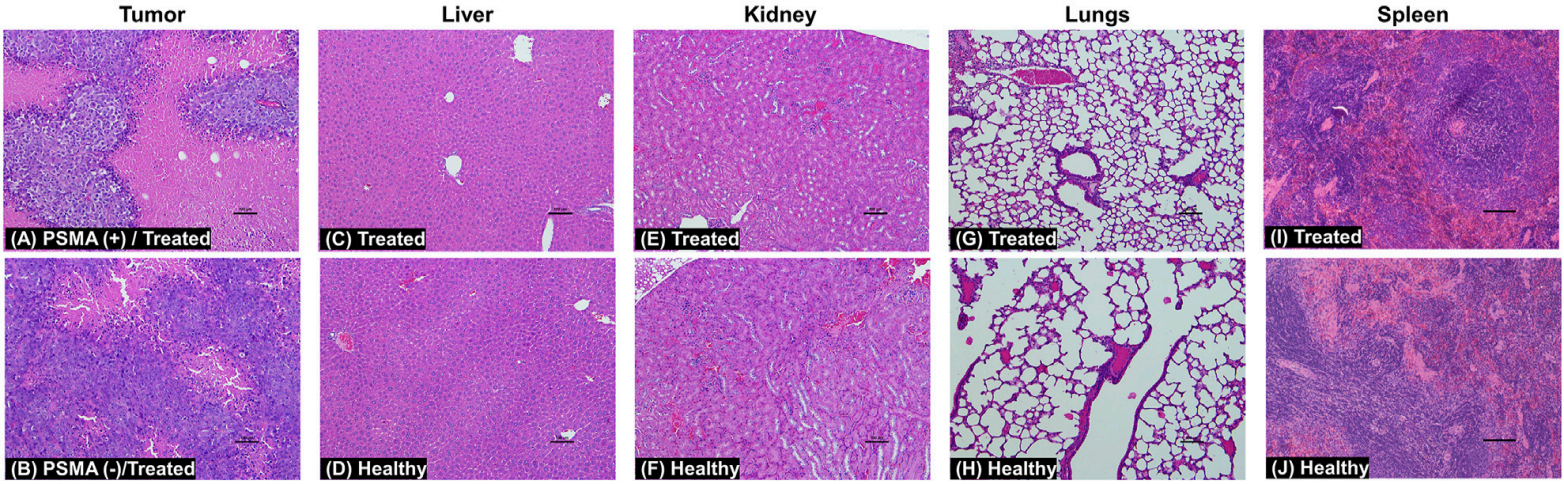
H&E staining images of tumors, liver, kidneys, lungs, and spleen at day 21. Treated mice were administered with a 5.0 mg/kg dose of 5D3(CC-MLN8237)_3.2_-CF750 or saline. Tissue sections reveal extensive necrosis in the PSMA (+) PC3-PIP tumors **(A)** and much less necrosis in the PSMA (−) PC3-Flu tumors **(B)** in treated mice. There was no toxicological damage in the liver **(C, D)**, kidney **(E, F)**, lung **(G, H)**, and spleen **(I, J)** tissues. (*n* = 4, magnification: ×10, scale bar: 100 µm).

To determine the toxicological clinical chemistry profile and CBC, we measured the levels of total T-Pro, BUN, ALP, Cre, and ALT levels, and RBC, WBC, and PLT counts in the blood. The results show a decrease in T-Pro and slight increase in Cre levels but these values were similar to healthy controls. The WBC and PLT counts in the blood of mice treated with 5D3(CC-MLN8237)_3.2_-CF750 was slightly decreased (Figure 7) likely due to consumption due to inflammation of the cancer and its necrosis. There is evidence of high liver uptake of the ATC in *ex vivo* optical imaging. However, all clinical chemistry and CBC changes are within the range of standard healthy levels. It is possible that ATC are not internalized into liver cells and show no significant liver toxicity since no liver enzymes were elevated. Supplementary Figure S8 shows the plot of the change in body weights of mice during the treatment period. No loss in body weight was observed in all mice of treated, untreated, and healthy groups. Overall, the toxicological study results are consistent with H&E staining of major organs isolated from mice following treatment. The H&E histological staining results show more necrosis in the PSMA (+) PC3-PIP tumor, less necrosis in the PSMA (−) PC3-Flu tumor, and no necrosis or other toxicological damage in the mice liver, kidneys, lungs, and spleen of 5D3(CC-MLN8237)_3.2_-CF750 treated group (Figure 8). Slides were reviewed in a blinded manner by a veterinary pathologist.

## 4 Conclusion

We have developed 5D3(CC-MLN8237)_3.2_, novel PSMA (+) PC targeting ATCs using anti-PSMA 5D3 mAb and Aurora A kinase inhibitor, MLN8237, conjugated via a TCO-Tz click chemistry-based stable linker (CC linker). The use of Aurora A kinase as a conjugated payload, and TCO-Tz click chemistry linker is demonstrated for the first time in the ADC development. 5D3(CC-MLN8237)_3.2_ were labeled with visible light or NIR fluorophores to integrate theranostic properties and to evaluate ATCs by *in vitro* and *in vivo* optical imaging. 5D3(CC-MLN8237)_3.2_ ATCs exhibited unaltered characteristics of 5D3 mAb in binding affinity, internalization, and subcellular localization. 5D3(CC-MLN8237)_3.2_-CF750 exhibited an enhanced therapeutic efficacy *in vivo* with minimal side effects and systemic toxicities in preclinical human PC xenograft mouse models compared to previously developed 5D3-based ADCs. The results indicate that 5D3(CC-MLN8237)_3.2_ ATCs could be valuable theranostics with potential benefit to patients with PSMA (+) PCs. To further prove these points, we are expecting to extend the therapeutic and toxicological studies to prostate cancer PDX models in the near future.

## Data Availability

The original contributions presented in the study are included in the article/Supplementary Material, further inquiries can be directed to the corresponding author.
