# Supplementary material for: Development and therapeutic evaluation of 5D3(CC-MLN8237)3.2 antibody-theranostic conjugates for PSMA-positive prostate cancer therapy
Source: Front Pharmacol. 2024 May 1;15:1385598. doi: 10.3389/fphar.2024.1385598 (PMC11094276; doi:10.3389/fphar.2024.1385598)
Supplement: Supplementary file 1 [file DataSheet1.DOCX]

Supplementary Material

Development and Therapeutic Evaluation of 5D3(CC-MLN8237)_3.2_ Antibody-Theranostic Conjugates for PSMA-Positive Prostate Cancer Therapy

Ioanna Liatsou^1^, Betelhem Assefa^1^, Wathsala Liyanage^2^, Sharmane Surasinghe^3^, Zora Nováková^4^, Cyril Bařinka^4^, Kathleen Gabrielson^5^, Venu Raman^1,6,7,8^, Dmitri Artemov^1,6^, Sudath Hapuarachchige^1,6*^

***Correspondence:** Sudath Hapuarachchige, Ph.D.

shapuar1@jh.edu


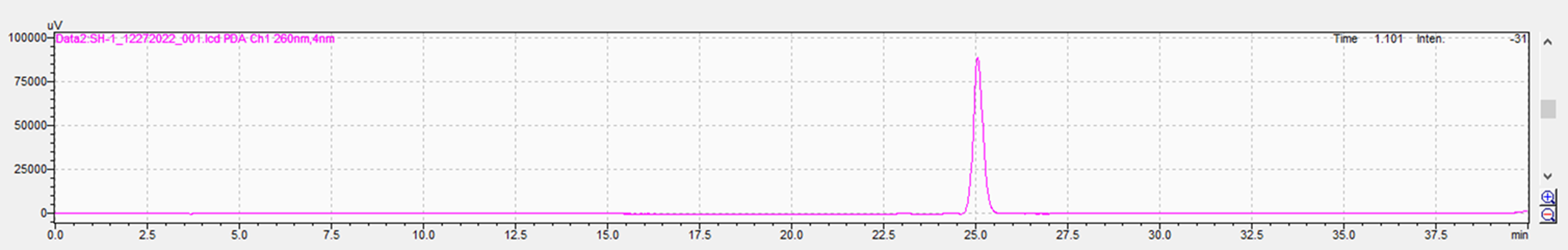
(a)

**Supplementary Figure 1.** HPLC spectrum of MLN8237-Tz. (a) chromatogram showing the purity of MLN8237-Tz.


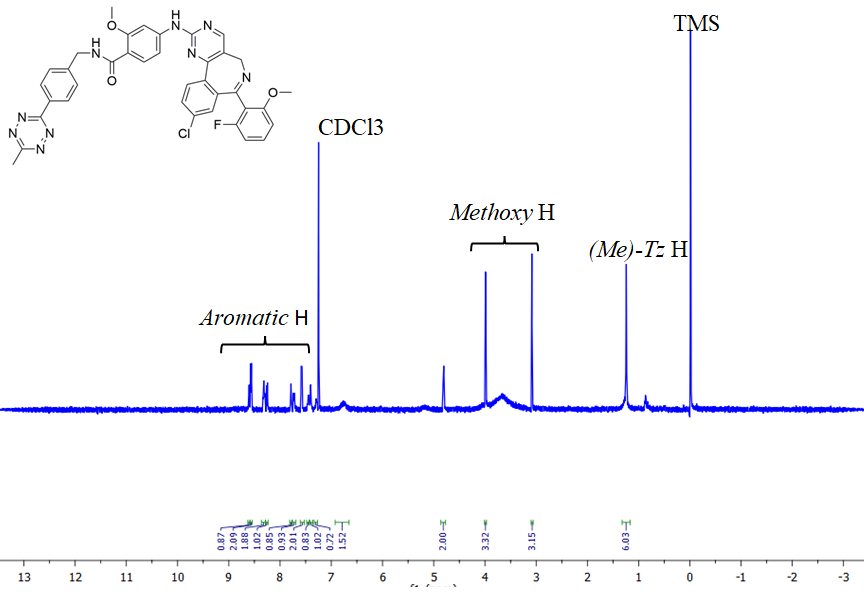


**Supplementary Figure 2.** ^1^H NMR of MLN8237-Tz


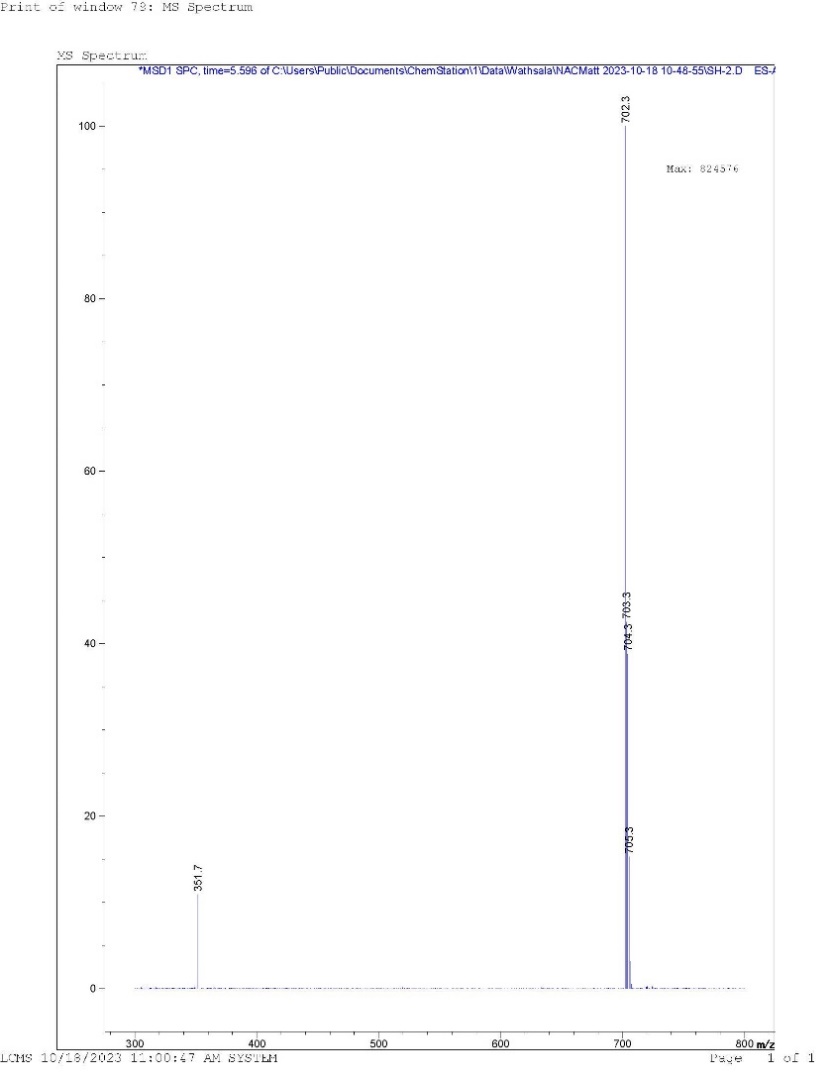


**Supplementary Figure 3.** The mass spectrum of MLN8237-Tz confirms the mass of the product.


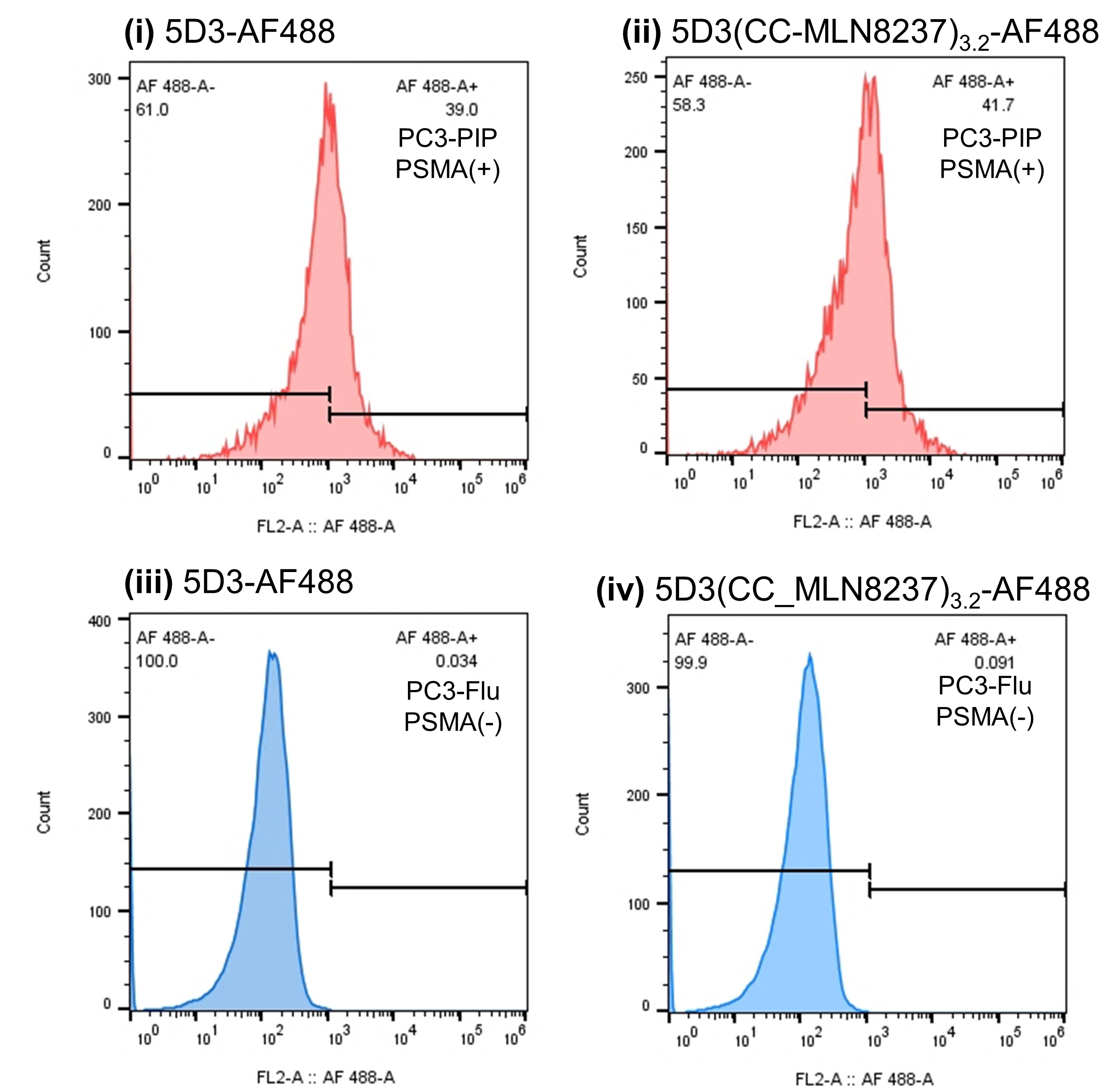


**Supplementary Figure 4.** Flow cytometry histogram showing unchanged affinity of (i) 5D3-AF488 and (ii) 5D3(CC-MLN8237)_3.2_-AF488 in PSMA(+) PC3-PIP cells and compared with the affinity of (ii) 5D3-AF488 and (d) 5D3(CC-MLN8237)_3.2_-AF488 in PSMA(-) PC3-Flu cells.


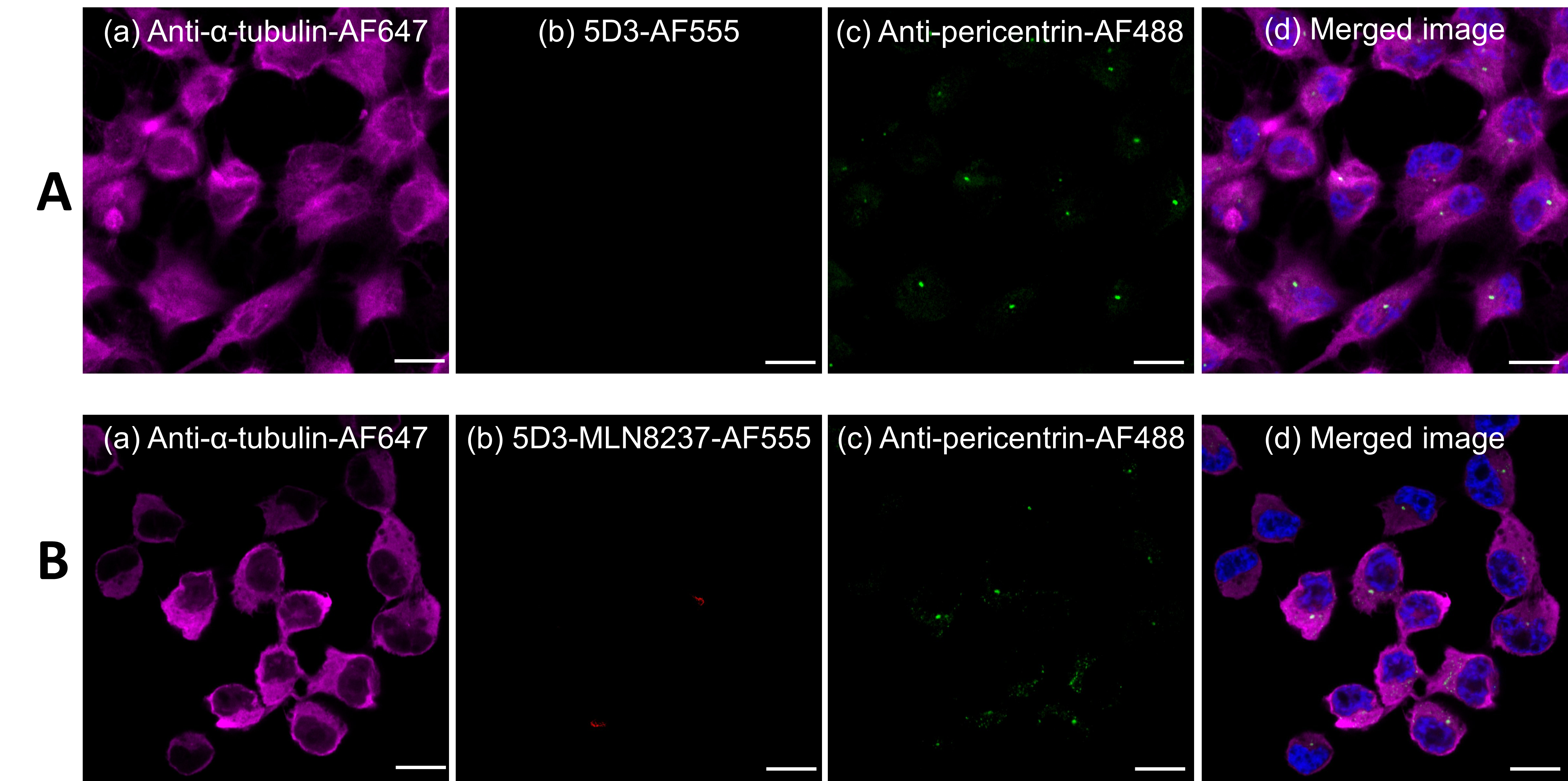


**Supplementary Figure 5.** Confocal fluorescence images of PSMA(-) PC3-Flu cells treated anti-α-tubulin-Alexa Fluor 647, anti-pericentrin Alexa Fluor 488 with 5D3-AF555. (a) Anti-alpha-tubulin (far-red channel), (b) 5D3-AF555 (red channel), (c) anti-pericentrin centrosome marker (green channel), and (d) merged image of all channels with the blue channel for Hoechst 33342 nuclei counterstaining. (Scale bar = 10 µM and magnification 63X).


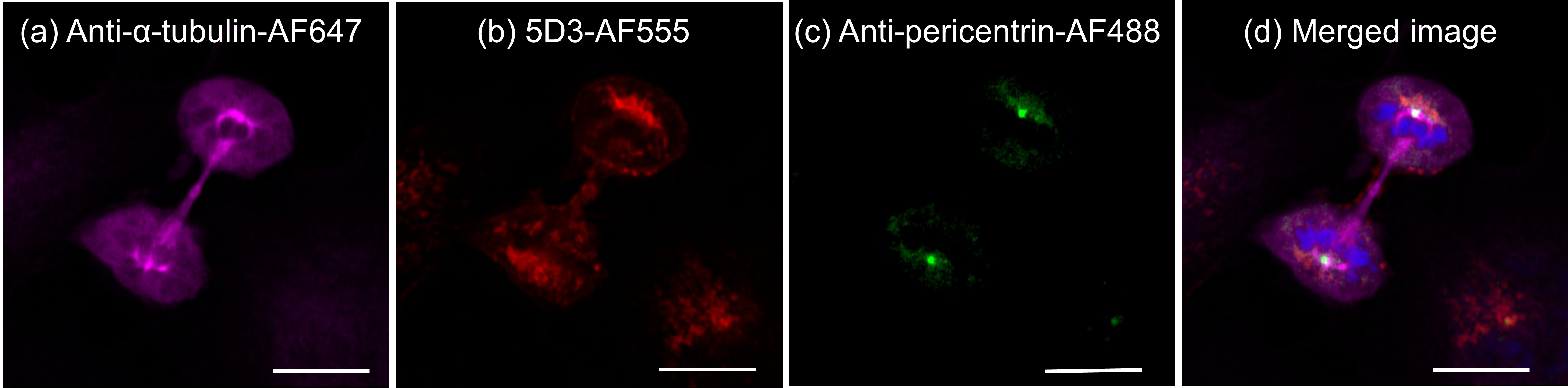


**Supplementary Figure 6.** Telophase of cell division in PC3-PIP cells showing the localization of 5D3 mAb at the centrosome. Cells treated with anti-α-tubulin-Alexa Fluor-647 (a, far-red channel), 5D3-MLN8237-AF555 (b, red channel), and anti-pericentrin Alexa Fluor-488 (c, green channel), with channel merged image with the blue channel for Hoechst 33342 nuclei counterstaining (d). (Scale bar = 10 µM and magnification 63X).


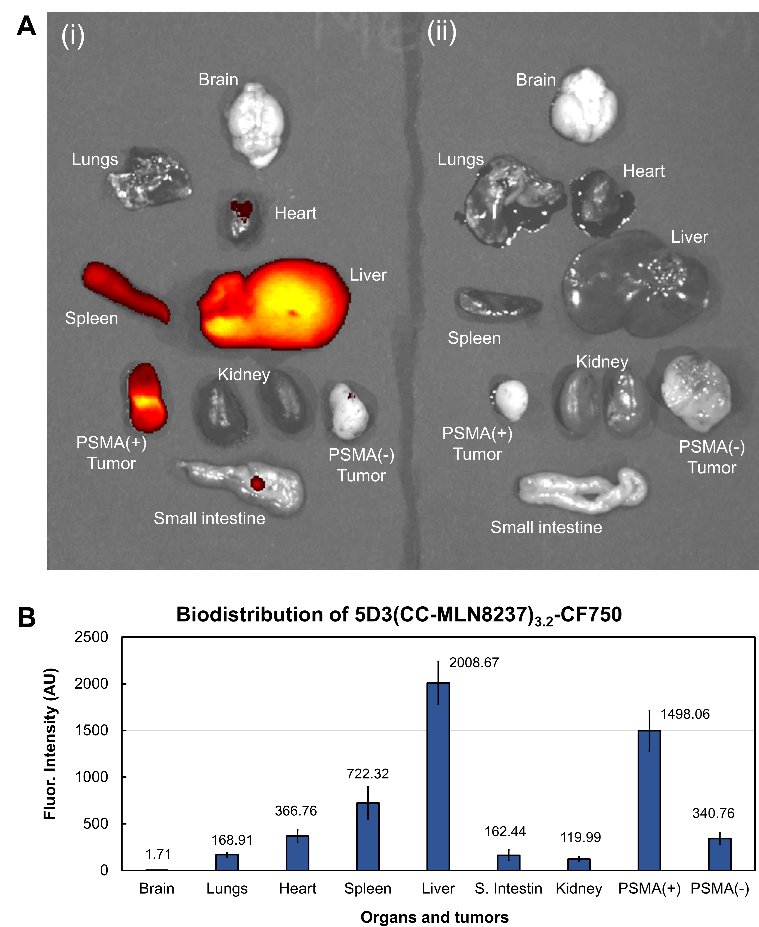


**Supplementary Figure 7.** Biodistribution of 5D3(CC-MLN8237)_3.2_-CF750 in mouse models. (A) *Ex vivo* fluorescence images of tumors and selected vital organs of mice treated with 5D3(CC-MLN8237)_3.2_-CF750 (i) and saline control (ii) were taken using Xenogen IVIS *in vivo* optical imaging system. (B) Quantitative analysis of 5D3(CC-MLN8237)_3.2_-CF750 biodistribution after 96 h (n=3).


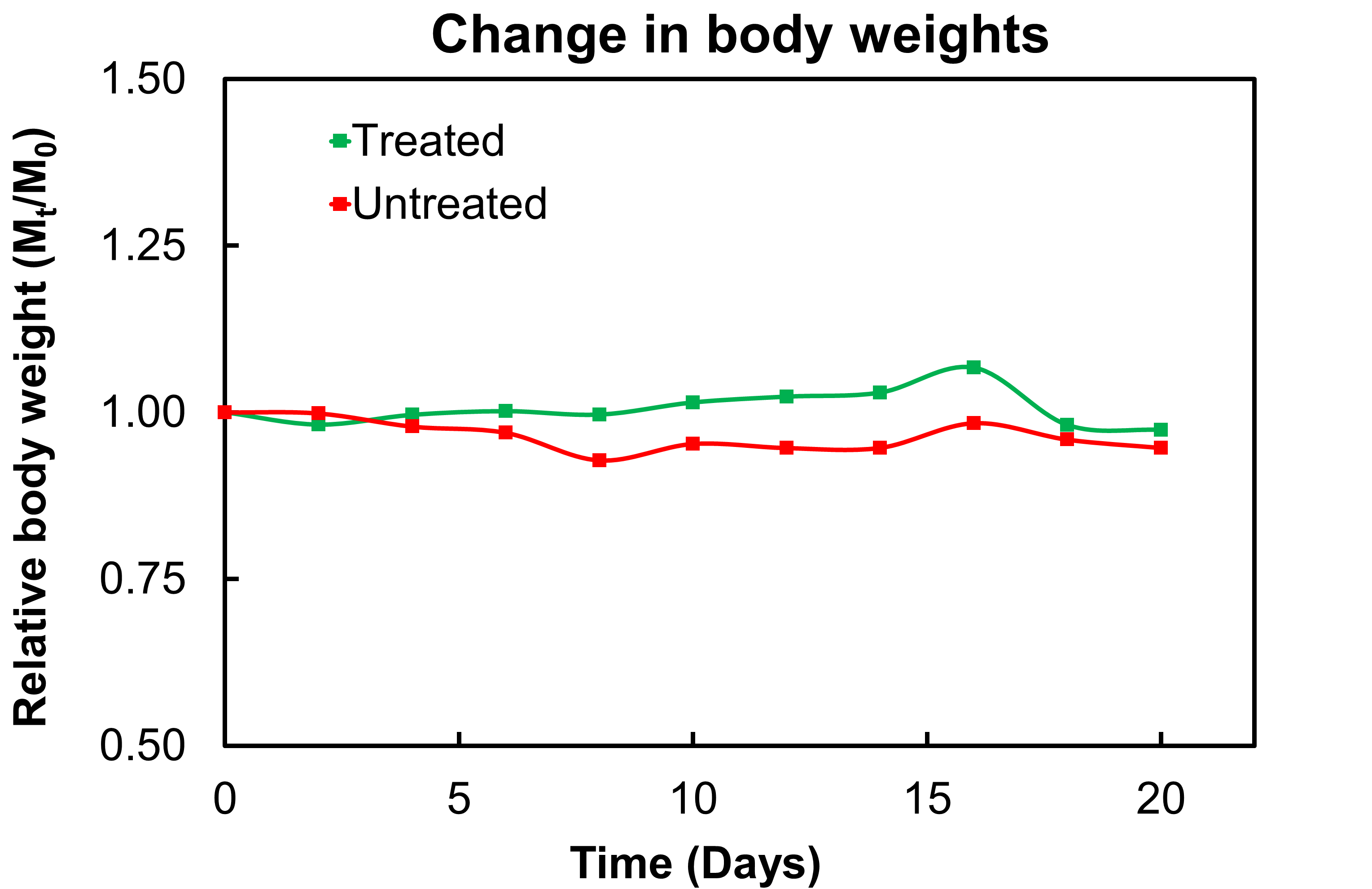


**Supplementary Figure 8.** Change in the animal body weight during the therapy relative to the initial body weight (n = 10/group).
